# Utilizing herbarium specimens to quantify historical mycorrhizal communities

**DOI:** 10.1002/aps3.1223

**Published:** 2019-02-28

**Authors:** J. Mason Heberling, David J. Burke

**Affiliations:** ^1^ Section of Botany Carnegie Museum of Natural History 4400 Forbes Avenue Pittsburgh Pennsylvania 15213 USA; ^2^ Department of Ecology and Evolutionary Biology University of Tennessee Knoxville Tennessee 37996 USA; ^3^ The Holden Arboretum 9500 Sperry Road Kirtland Ohio 44094 USA; ^4^ Department of Biology Case Western Reserve University Cleveland Ohio 44106 USA

**Keywords:** arbuscular mycorrhizal fungi, *Arisaema triphyllum*, forest understory, herbarium specimens, *Maianthemum racemosum*, *Trillium* spp

## Abstract

**Premise of the Study:**

Mycorrhiza are critical to ecosystem functioning, but a lack of historical baseline data limits our understanding of the long‐term belowground effects of global change. Herbarium specimens may provide this needed insight. However, it is unknown whether DNA of arbuscular mycorrhizal fungi (AMF) can be reliably extracted from vascular plant specimen roots.

**Methods:**

We sampled roots from herbarium specimens of four herbaceous forest species collected in western Pennsylvania between 1881–2008. Using molecular methods (terminal restriction fragment length polymorphism and sequence analysis), we quantified AMF communities from specimen roots and tested for contamination.

**Results:**

We successfully amplified AMF DNA from 44% (21/48) of the root but not leaf samples, indicating specimen contamination was negligible. As expected, there were significant differences in AMF composition between plant species (*P* < 0.05). However, no differences in AMF communities were detected through time, possibly due to limited sample size and low amplification rates in recent collections.

**Discussion:**

Herbaria have potential as sources of valuable belowground microbial data to answer questions across geographic, temporal, and taxonomic scales otherwise not feasible. Ongoing methodological developments will only magnify this potential. Further tests are needed to determine curatorial practices that maximize this innovative use of herbarium specimens.

Belowground soil communities, especially fungal symbionts, play critical roles in ecosystem responses to global change factors such as land use changes, increased temperatures, elevated atmospheric CO_2_, drought, soil acidification, nitrogen deposition, and non‐native species invasions (Egerton‐Warburton and Allen, 2000; Wolf et al., 2003; Treseder, 2004; Wolfe and Klironomos, 2005; Kivlin et al., 2013; Allen et al., 2014; Mohan et al., 2014; Carrino‐Kyker et al., 2016). However, our understanding of how mycorrhizal communities respond to chronic human‐mediated environmental changes primarily depends upon short‐term observations or fertilization/warming experiments (most studies ≤1 year in duration; Mohan et al., 2014). Therefore, we lack adequate baseline data to assess how belowground soil communities respond to the chronic environmental disturbances that define the Anthropocene. Here, using deciduous forest understory herbaceous perennials as a case study, we tested whether mycorrhizal DNA stored in herbarium specimen roots might provide these needed data.

Arbuscular mycorrhizal fungi (AMF) in the phylum Glomeromycota are pervasive in deciduous forest species, forming associations with the roots of ca. 74% of all angiosperm species in this habitat (Brundrett, 2009). The majority of herbaceous species in deciduous forest understories rely upon AMF to obtain water and nutrients (Brundrett and Kendrick, 1988). These root symbionts form complex associations with their plant hosts (Brundrett, 2004), often involving multiple taxa of fungal partners to form taxonomically and functionally diverse microbial communities in the rhizosphere that are still being understood. Therefore, in deciduous forests, where most diversity comprises AMF‐dependent species (Brundrett and Kendrick, 1988; Whigham, 2004; Gilliam, 2007), a mechanistic understanding of plant dynamics from individual to ecosystem scales inherently requires a detailed understanding of microbial interactions belowground. Furthermore, understory species in the herbaceous layer in temperate deciduous forests are increasingly threatened by human activities (Gilliam, 2007). However, the ecology and life history of few understory herb species have been studied in detail, including their interactions with AMF, limiting our ability to conserve these species in the face of environmental change (Whigham, 2004).

Given the importance of AMF for growth, survival, and reproduction in forest herbs, population declines in these species may be mediated by the unseen changes in belowground communities as a result of human activities, but the quantification of long‐term responses to global change by soil fungi is difficult. As a result, belowground responses are either neglected or a major source of uncertainty in models of global change (Johnson et al., 2013). Experimental field manipulations have found significant decreases in mycorrhizal abundance with nitrogen and phosphorus fertilization, and increases with elevated atmospheric CO_2_ (Treseder, 2004). However, our current understanding is limited to short‐term studies that experimentally manipulate one or several factors (e.g., CO_2_, nitrogen, water). Although these experiments are useful to understand the acute responses of mycorrhiza to key components of global environmental change, it remains unclear how short‐term experimental manipulations compare to the combined effects of chronic global change stressors these communities have experienced over the past century (Allen et al., 2014). In a unique study using archived soil samples collected from 1937–1999, Egerton‐Warburton et al. (2001) found significant shifts in AMF species composition in southern California, USA, as a result of anthropogenic nitrogen deposition. Because archived soil samples are limited in scope, similar retrospective analyses are rare.

Herbaria might serve as an unrealized source of belowground microbial data to enhance our understanding of mycorrhiza, their distributions, their taxonomic and biogeographic affinities toward plant host species, and their responses to environmental change across space and time. Collected by many thousands of botanists over the past four centuries, nearly 390 million specimens of preserved plants, fungi (especially macrofungi), algae, and related taxa currently reside in more than 3000 herbaria across the world (Thiers, 2018). Modern herbaria were primarily established to function as resources for taxonomy, floristics, and species identification. Although these traditional, anticipated roles remain critical botanical research, herbarium specimens and their associated data have been recently leveraged in novel ways unanticipated by the original collectors (Heberling and Isaac, 2017). These rapidly expanding innovative uses for specimens include (to name a few): uncovering phenological responses to climate change (Willis et al., 2017), tracking industrial pollution (Rudin et al., 2017), quantifying morphological change through time (Buswell et al., 2011), and extracting DNA for systematics and genetic study (Bieker and Martin, 2018). The potential use of herbarium specimens to understand belowground biology and mycorrhizal response to global change remains unexplored. Vascular plant specimens frequently include belowground structures (including roots), especially in certain species. To date, however, herbarium specimen roots have been narrowly used as a taxonomic character in certain species groups (e.g., belowground morphology, type of perennial storage organ, presence of nodules).

Here, we applied well‐established molecular methods to test whether herbarium specimen roots can be used to quantify taxonomic, temporal, and geographic patterns in AMF communities. We sampled root tissue from herbarium specimens of four common forest understory herbaceous perennials known to associate with AMF that were collected in the Greater Pittsburgh Region, Pennsylvania, USA, over the past 135+ years. Our goals were to determine (1) whether fungal DNA could be extracted from the root samples and amplified with PCR for downstream analysis of fungal community identity and community composition, (2) whether specimen age affects the ability to extract and amplify DNA, and (3) whether the method could be used to determine differences in the AMF community between plant species. Furthermore, we sought to use these results as a proof of concept to assess whether this novel herbarium approach can be applied in future studies at broader scales to determine whether AMF community composition has changed through time as a result of factors of anthropogenic global change.

## METHODS

### Sampling

We sampled roots from 48 plant specimens collected between 1881–2008 and archived in the Carnegie Museum herbarium (CM). We sampled both root and leaf tissue from 12 plants each of four focal species: *Arisaema triphyllum* (L.) Schott, *Maianthemum racemosum* (L.) Link, *Trillium erectum* L., and *T. grandiflorum* (Michx.) Salisb. (Appendix 1). These species were selected because they are important components of forests in the region and are extensively colonized by mycorrhizal fungi, with a previous study finding total root length colonized ranging from 39% to 94% in these species (Burke, 2008). For each species, we surveyed all CM specimens collected in Allegheny and adjacent counties in southwestern Pennsylvania, USA. We chose specimens that had sufficient root tissue such that our destructive sampling would not compromise future use of the specimen. To control for any confounding effects based on season the specimens were collected (e.g., spring vs. summer), we further narrowed these specimens to those that shared the same phenological stage (flowering). All samples were collected during peak flowering but before fruit set (generally April and May for these spring‐blooming, summer‐green species). For temporal replication, three specimens of each species were sampled from each of the following predefined four time periods: (1) 1870–1910, (2) 1911–1950, (3) 1951–1990, and (4) 1991–2008. In total, 48 specimens were included in our analysis (3 specimens × 4 species × 4 time periods). All specimens were imaged prior to any sampling (specimen images available at http://www.midatlanticherbaria.org).

Sampling was conducted using forceps and razor blades that were sterilized before use to avoid cross‐contamination. A unique, pre‐sterilized set of forceps and blade was used for each specimen and tissue type (root and leaf). We avoided any glue used to mount specimens. Sampling along the length of the specimen to ensure diversity of root ages, four complete roots per specimen were excised at the rhizome/corm (approximately 10 cm of root per specimen collected). Samples were placed in sterile, 2‐mL cryogenic vials (Thermo Fisher Scientific, Waltham, Massachusetts, USA) and tightly sealed. At time of root sampling, one 0.2‐cm^2^ piece was removed from each of two leaves to test for specimen contamination (i.e., leaf tissue should not have AMF DNA). Because AMF spores are generally not airborne, we suspected herbarium specimen contamination as a result of dust accumulation to be unlikely. However, we sampled leaves along with roots to test for contamination. In the absence of contamination, AMF should be found on roots but not leaves.

### DNA extraction and amplification of AMF

**Figure 1:**
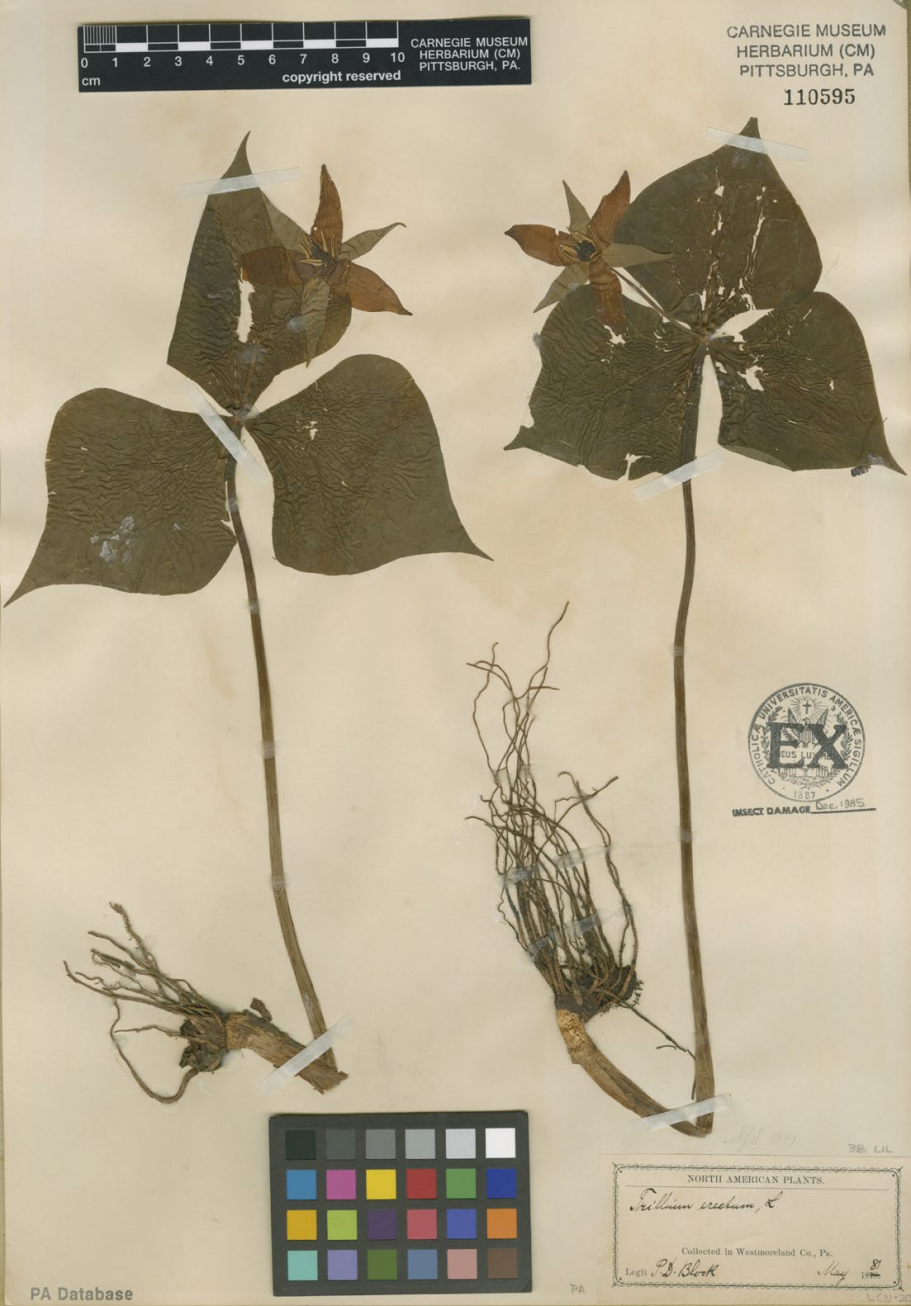
Representative herbarium specimen sampled in the current study (*Trillium erectum*; May 1881; *P.D. Block s.n*. [CM110595]; Westmoreland County, Pennsylvania, USA). Note that specimen roots and associated soil frequently come into contact with leaves during collection, and some specimens are pressed such that roots physically contact leaves on the herbarium sheet. It is likely that leaves come into contact with soil originally from the rhizosphere of the same specimen.

DNA was extracted from one‐half of the collected leaf and root tissue. During our initial work, we found that leaf tissue frequently amplified with AMF‐specific primers (results not shown), suggesting leaf tissue may be contaminated with AMF DNA. It is unclear if this contamination occurred at the time of collection, specimen processing (e.g., drying, mounting to herbarium sheet), decades of specimen handling, or long‐term storage in herbarium cabinets. However, we suspect AMF DNA present on specimen leaves originated from the same specimen because many samples had the stems bent such that root tissue was near or in contact with the leaves of the specimen (Fig. 1). Furthermore, soil is often on plant leaves in the field, and collection practices in the field do not control for leaf contamination. Consequently, we adopted a sample washing approach to eliminate contamination of AMF on leaf and root tissues. Prior to DNA extraction, samples were soaked in 500 μL of sterile PCR‐grade water for 2 min. Regardless of whether true external contamination was present or not, this protocol ensured that any AMF amplification from specimen roots reflected AMF within the tissue rather than stray contamination from other sources. However, this conservative approach removed soil adhered to the roots, an important component of the rhizosphere that contains AMF.

Washed samples were then transferred into a 1.5‐mL bead‐beating tube containing 300 mg of 400 μM sterile glass beads (VWR, West Chester, Pennsylvania, USA), 200 mg of 1‐mm sterile glass beads (Chemglass, Vineland, New Jersey, USA), and 750 μL of 2% cetyltrimethylammonium bromide (CTAB). Samples were bead beaten using a Precellys homogenizer (Bertin Technologies, Montigny‐le‐Bretonneux, France) for 80 s and purified using phenol‐chloroform extraction (Burke, 2008; Hewins et al., 2015). DNA was precipitated using 20% polyethylene glycol 8000 in 2.5 M NaCl, and DNA was suspended in 50‐μL Tris EDTA (TE) buffer and stored in a 1.5‐mL low‐retention microcentrifuge tube (Thermo Fisher Scientific) at −20°C until analysis. Sample blanks that consisted only of sterile PCR‐grade water were also used and carried through the extraction process to control for laboratory contamination. DNA quality was assessed through gel agarose electrophoresis using the Invitrogen Low DNA Mass Ladder (Thermo Fisher Scientific). See Appendix S1 for gel example.

To examine the AMF community, we targeted the 18S rRNA gene using primers NS31 (Simon et al., 1992) and AM1 following general procedures in Helgason et al. (1998). PCR was carried out in 50‐μL reaction volumes using 2 μL of the purified DNA, 2 units GoTaq DNA Polymerase (Promega Corporation, Madison, Wisconsin, USA), 1.0 μL of each primer (10 mM), 2.5 μL of bovine serum albumin (10 mg/mL), and 4.0 μL of MgCl (25 mM) on a PTC 100 Thermal Cycler (MJ Research, Boston, Massachusetts, USA). Primers were labeled with fluorochromes 6FAM (AM1) and HEX (NS31) for community analysis using terminal restriction fragment length polymorphism (TRFLP). For TRFLP, restriction digest was carried out using the endonucleases *Hin*fI and *Hsp*92 (Promega Corporation) in separate reactions (i.e., two restriction digests per sample). We have used these primers and conditions for several years with good results for analysis of AMF communities (Burke, 2008; Burke et al., 2011). Although it is possible that these primers could miss some AMF taxa, in our experience with the plant species examined here, these primers are specific for AMF and do not co‐amplify plant DNA, which can happen with some primer sets and species. Based on our previous experience, the NS31/AM1 primers work well for the four species included in this study, showing high specificity for the AMF DNA. TRFLP was completed through Cornell University's Biotechnology Resource Center using an Applied Biosystems 3730xl DNA Analyzer and PeakScanner software version 1 (Applied Biosystems, Foster City, California, USA). TRFLP is considered a cost‐effective community profiling method that compares favorably to next‐generation sequencing for examination of community response to environmental conditions (Camarinha‐Silva et al., 2012; van Dorst et al., 2014).

To confirm that AMF amplification from herbarium roots was successful, we constructed a clone library of AMF from *T. erectum* root amplicons. PCR was conducted as described above on *T. erectum* root samples using unlabeled primers AMG1F and AM1 (Hewins et al., 2015), and PCR product was gel purified using the Wizard SV Gel and PCR Clean‐Up System (Promega Corporation). We used these primers because we found that they amplify a wide range of AMF without amplifying the DNA of herbaceous forest plants and could be used for future real‐time PCR (qPCR) assessments of AMF root colonization (see Hewins et al., 2015). We used the QIAGEN PCR Cloning Plus kit (QIAGEN, Valencia, California, USA) to develop a library of 48 clones for sequencing. Plasmids from selected colonies were grown overnight in Luria–Bertani media and then purified with the Wizard Plus SV Minipreps DNA Purification System (Promega Corporation) following the manufacturer's protocol. Cloned AMF rDNA were sequenced (Sanger sequencing) using the BigDye Terminator version 3.1 Cycle Sequencing Kit (Applied Biosystems). Sequencing was conducted at Cornell University's Biotechnology Resource Center on an Applied Biosystems 3730xl DNA sequencer. The identification of AMF species was determined using the BLAST tool through the National Center for Biotechnology Information (NCBI).

### Statistical analyses

We assessed whether AMF communities differed among plant species or across collection time. We used nonmetric multidimensional scaling (NMS) procedures using PC‐ORD 4 (MjM Software, Gleneden Beach, Oregon, USA) to quantify and visualize community differences. The Sørenson distance was used with a random starting configuration and included 250 runs with real data, 250 runs with randomized data, and a Monte Carlo test–selected final dimensionality. If additional dimensions of the ordination did not further reduce stress by 5 or more, it was not considered to have improved the ordination and the highest dimensionality that met this criterion was used for the final ordination. We chose the Sørenson distance for NMS because it has been shown to be an effective measure of similarity between samples (McCune and Grace, 2002). TRFLP peak area was used for these analyses. However, peaks that comprise less than 1% of the total TRFLP profile area are often not repeatable between replicate profiles, so only those peaks >1% of the profile area were included (Burke and Chan, 2010). We used terminal restriction fragments generated with both restriction enzymes in the same analysis. TRFLP profiles from both restriction enzymes were combined for the analysis. TRFLP data were organized and sorted prior to analysis using the program TRFLPR (Petersen et al., 2015). Proportional abundance of detected TRFLP peaks was used for NMS, and all proportional abundance data were arcsin–square root transformed before analysis (Burke, 2008). To test whether measured AMF communities differed by host plant species and/or time of collection, we performed a two‐way permutational multivariate ANOVA (PERMANOVA) on the distance matrix with time period of collection and species as predictors using the *adonis* function in the vegan package in R (Oksanen et al., 2018).

## RESULTS

### Amplification of AMF from herbarium specimens

**Figure 2:**
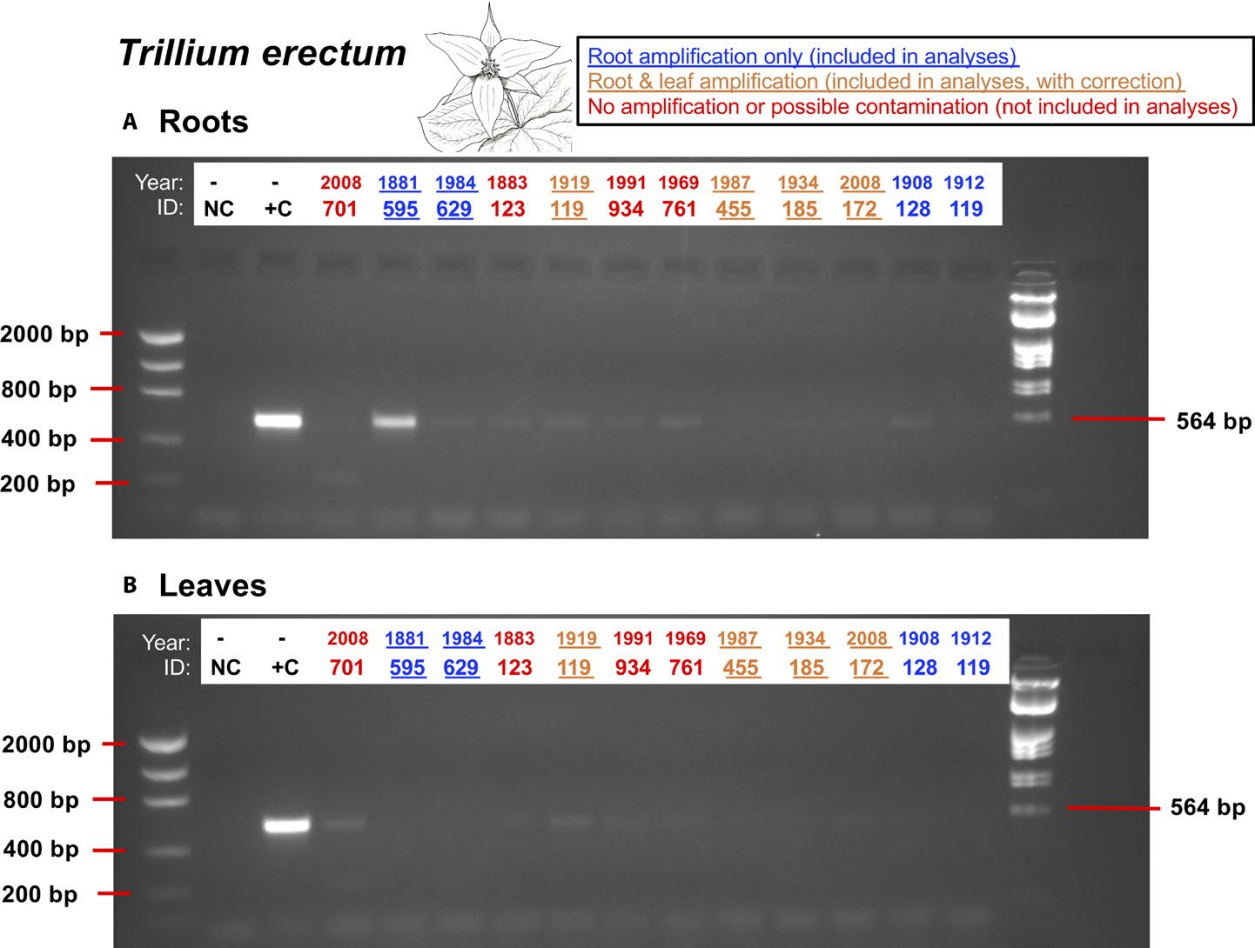
PCR results for *Trillium erectum* samples as run into a 2% agarose gel in TAE buffer. Samples are flanked by DNA markers that indicate size. Invitrogen Low DNA Mass Ladder is on the left and λ/*Eco*R1 + *Hin*dIII ladder is on the right (Thermo Fisher Scientific), with fragment sizes shown. NC = negative control, +C = positive control arbuscular mycorrhizal fungi (AMF) plasmid. ID numbers indicate the last three digits of the specimen ID number (see Appendix 1). Note: two specimen ID numbers end in 119; the column labeled 119 on the far right corresponds to CM023119. Year refers to year of collection. Blue underlined labels indicate samples with root amplification that were included in analyses. Orange underlined labels indicate samples that showed some AMF amplification in leaves, which were removed from root profiles prior to community analysis. Labels in red indicate samples that were not included in analyses either because roots failed to amplify or leaf TRFLP profiles suggested contamination.

**Figure 3:**
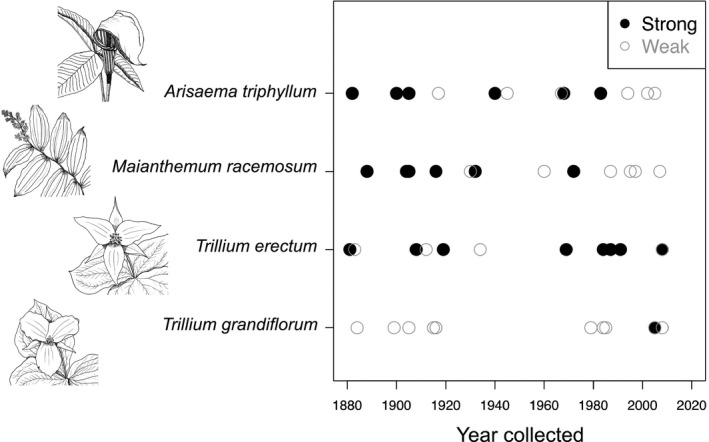
Success of arbuscular mycorrhizal fungi (AMF) DNA extraction and amplification from roots and year of collection. Twelve specimens per species were sampled. “Strong” designates a specimen that amplified successfully for AMF, and “Weak” designates a specimen that did not amplify for AMF.

After washing in sterile PCR‐grade water, 44% (21/48) of the root samples amplified for AMF, although success rates varied by species. *Trillium erectum* had the highest success rate, with eight of the 12 samples amplified for AMF (Fig. 2, Appendix 1). However, even after washing in sterile PCR‐grade water, some *T. erectum* leaf samples had low levels of AMF amplification (Fig. 2). Because AMF DNA amplification in leaves might indicate external contamination, we applied a correction in community analysis (described below). Low levels of leaf amplification were probably due to within‐specimen cross‐contamination from roots, because many herbarium plant stems were bent such that roots were near or in contact with leaf tissue (Fig. 1). Nonetheless, leaf tissue served as a useful negative control to determine whether the AMF we amplified emanated from the herbarium root samples or general contamination from external sources (e.g., dust). Both *A. triphyllum* and *M. racemosum* samples were similarly successful, with 50% (6/12) of root samples amplifying for each species without any associated leaf amplification (*A. triphyllum*, Appendix S2; *M. racemosum*, Appendix S3). Our amplifications were least successful in *T. grandiflorum*, with only one of 12 samples amplifying with PCR (Fig. 3, Appendix 1). Therefore, given this low sample size, we lumped the *Trillium* L. species together for subsequent community analysis (see below).

Sample amplification was not limited by specimen age. We routinely amplified samples that were more than 100 years old (Fig. 3). The oldest sample we amplified was a *T. erectum* specimen collected in 1881 (CM110595). In fact, this sample was one of the strongest amplifications overall, and leaf amplification was not detected at all for this sample (Fig. 2). Paradoxically, the oldest samples were more likely to amplify than newer samples (Fig. 3): 67% (8/12) of the oldest age class (1870–1910) amplified, 33% (4/12) of the second oldest age class (1911–1950), 50% (6/12) of the second youngest age class (1951–1990), and only 25% (3/12) of the youngest samples (1991–2008) amplified.

We recovered a diverse group of AMF through sequence analysis (Table 1). Recovered sequences were deposited in GenBank (accession numbers MH800915–MH800957). Of 48 clones, 43 clones were good matches to AMF taxa; two clones did not sequence properly and three were high matches (e.g., 99%) to Basidiomycetes. Recovered AMF clones included taxa in the genera *Glomus*,* Funneliformis*, and *Rhizophagus*. Although our species library was limited, it revealed a diversity of AMF taxa and the specificity of the method for this group. This confirms that PCR product from herbarium root samples originated from AMF DNA associated with the roots.

**Table 1:** Arbuscular mycorrhizal fungi (AMF) clones recovered from herbarium root samples in the current study using primers AMG1F and AM1 (see Hewins et al., 2015).^a^

Clone ID no. (Accession no.)	No. of clones recovered	Best description based on NCBI matching^b^	Identity, %	Accession no. of best match
46 (MH800955)	1	*Glomeraceae* sp. clone MR043.b small subunit ribosomal RNA gene, partial sequence	99	KY234440.1
1 (MH800915)	1	*Glomeromycotina* sp. isolate T333 small subunit ribosomal RNA gene, partial sequence	99	MG576070.1
12 (MH800924)	1	*Glomus caledonium* strain E4 18S ribosomal RNA gene, partial sequence	94	HQ588777.1
16 (MH800928)	1	*Glomus intraradices* partial 18S rRNA gene, BEG123	88	AJ505617.1
17 (MH800929)	6	*Glomus mosseae* partial 18S rRNA gene, clone AZ225C/2‐52	98	FR751309.1
3 (MH800917)	3	*Glomus perpusillum* isolate OTU68 small subunit ribosomal RNA gene, partial sequence	99	MH286000.1
33 (MH800943)	1	*Glomus* sp. 2505.08.Otto 18S ribosomal RNA gene, partial sequence	99	AF480152.1
37 (MH800947)	1	*Glomus* sp. C/3‐10 partial 18S rRNA gene, clone C/3‐10	99	FR715050.1
2 (MH800916)	5	*Glomus* sp. Glo8 18S rRNA gene	98	AJ309464.1
32 (MH800942)	1	*Glomus* sp. IMA1/3‐9 partial 18S rRNA gene, clone IMA1/3‐9	97	FR773835.1
7 (MH800921)	5	*Glomus* sp. isolate AM10 18S ribosomal RNA gene, partial sequence	98	KX584362.1
14 (MH800926)	1	*Rhizophagus* cf. *irregularis* Att1225‐1 partial 18S rRNA gene, clone WD313‐1‐1	99	FR750223.1
47 (MH800956)	1	*Rhizophagus irregularis* strain A4 clone c2 18S ribosomal RNA gene, partial sequence	99	KY436249.1
4 (MH800918)	15	Uncultured *Sclerocystis* isolate CARPO_23 small subunit ribosomal RNA gene	100	MH150861.1

a Of 48 clones, two did not sequence properly and three were high matches (e.g., 99%) to other non‐AMF taxa. Forty‐three clones were good matches to AMF taxa as determined using the BLAST tool through the National Center for Biotechnology Information (NCBI). BLAST matching completed 11 October 2018. The recovered sequence for the clone type is shown along with the GenBank accession number. The number of clones matching the NCBI description is also indicated. The clone ID number is a representative clone for that sequence group; the number of clones recovered in that sequence group is shown in the second column.

b *Glomus intraradices* is currently named *Rhizophagus irregularis. Glomus mosseae* is currently named *Funneliformis mosseae*. Best description reflects NCBI taxa identification.

### AMF community analysis

Although many root samples amplified without corresponding amplification of leaf tissue, leaf tissue weakly amplified for some samples (e.g., *T. erectum*; Fig. 2). In these cases, we performed a correction before community analysis. In effect, leaf samples that amplified were also subject to TRFLP analysis, and any peaks present within the leaf profiles present within corresponding root profiles were deleted from the root profiles before analysis. This conservative approach could result in AMF taxa present in root tissue being removed from community analysis. However, it guarantees that community analysis only includes taxa that can be positively attributed to root association and not contamination from other sources (in the field, herbarium, or both). Correction of this kind was needed for some *T. erectum* samples, but *A. triphyllum* and *M. racemosum* root samples did not require correction as leaf tissue did not amplify.

**Figure 4:**
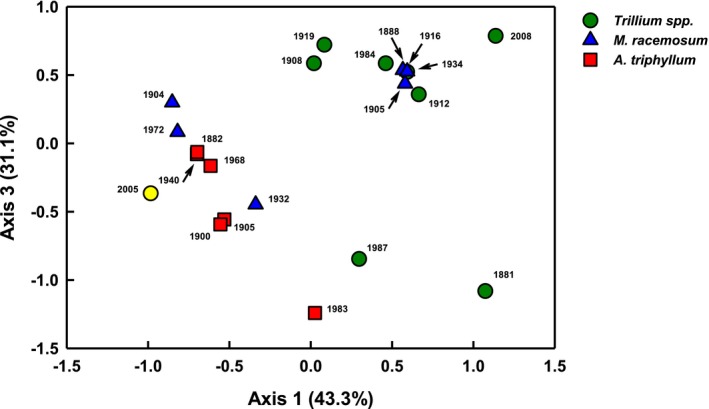
Nonmetric multidimensional scaling (NMS) ordination showing relationship between arbuscular mycorrhizal fungal (AMF) communities amplified from herbarium root samples for forest herbaceous species: *Arisaema triphyllum*,* Maianthemum racemosum*, and *Trillium* spp. (includes *T. erectum* and one sample for *T. grandiflorum*). Final stress was 8.4 for 21 samples that amplified for AMF (six *A. triphyllum*, six *M. racemosum*, and nine *Trillium* spp.). Yellow circle point represents *T. grandiflorum*.

Using this approach, we found that AMF community composition significantly differed by species (PERMANOVA, *F* = 2.455, *df* = 1, *P* = 0.04; Table 2). The results did not differ qualitatively with the removal of the single *T. grandiflorum* sample (Appendix S4). *Arisaema triphyllum* samples were tightly clustered, whereas samples for the other two species were more broadly distributed in NMS space (Fig. 4). NMS axes 1 and 3 cumulatively explained 74.4% of the variance, showing separation by host plant species in AMF community composition. NMS and PERMANOVA analyses indicate no significant effect of time period of collection on AMF community composition.

**Table 2:** Summary statistics for two‐way permutational multivariate ANOVA (PERMANOVA) of arbuscular mycorrhizal fungal communities among host plant species. The Bray–Curtis distance metric was used to calculate dissimilarity matrix

Source of variation	*df*	SS	MS	*F*	*P*
Species	1	0.3807	0.38074	2.455	0.0438*
Time	1	0.1265	0.12651	0.81577	0.5328
Species × time	1	0.1765	0.17652	1.13824	0.3432
Residuals	17	2.6365	0.15509		
Total	20	3.3202			

Note

*df* = degrees of freedom; *F* = test statistic; MS = mean squares; SS = sum of squares.

* Significant results (*P* < 0.05).

## DISCUSSION

Our core motivation in this study was to determine whether fungal DNA could be reliably extracted and amplified from dried roots of herbarium specimens. Using species with known AMF‐dependency and well‐developed primers (Burke, 2008; Burke et al., 2011; Hewins et al., 2015; Carrino‐Kyker et al., 2016), we successfully extracted and analyzed AMF DNA from specimens dating from 1881 (137 years old) to 2005, with a relatively high success rate (44% of sampled specimens). These results support the utility of using herbarium specimens in retrospective analyses of root symbionts. In addition, our results serve as a proof of concept that confirms the potential of herbaria as rich sources of belowground data for more detailed studies in the future. Although we were limited by sample size, our approach could be applied more widely to test the impacts of anthropogenic environmental change on both AMF community composition and the dynamics of their complex associations with plant species through space and time.

### Are herbaria an unrealized resource to quantify AMF community changes over the past century of environmental change?

Our results demonstrate that herbarium specimens have the potential to be utilized to effectively reconstruct historical AMF communities. However, given the scope of the current study, future work is needed to address specific global change hypotheses. This approach holds great promise in determining whether certain AMF taxa have declined, whether overall AMF diversity in the rhizosphere has declined, or even whether AMF associations have shifted among plant species.

Despite this potential, we found that more than half (56%) of the specimens we sampled did not successfully amplify. This success rate is not surprising, as degradation of DNA in historical samples is a problem (Pääbo et al., 2004; Gilbert et al., 2005) and low‐quality DNA may have interfered with amplification of some samples in our study. In addition, surface contamination of historical samples can interfere with amplification and analysis of ancient DNA. Decontamination steps, including harsh methods such as washing with bleach, are often required to eliminate surface contamination from some samples (e.g., see Lendvay et al., 2018 for decontamination of fossil wood with pollen). With our herbarium samples, more gentle decontamination with PCR‐grade water was sufficient to achieve decontamination of most root samples relative to leaf samples in our study. However, additional molecular approaches, such as nested PCR, could be used to improve amplification performance of herbarium specimens. Future work should also utilize high‐throughput (next‐generation) sequencing.

We expected that microbial DNA amplification in roots sampled from older specimens would be less successful due to time‐related DNA degradation. Surprisingly, we had greater success with older specimens compared to those collected after 1990. Two‐thirds of specimens collected before 1910 were successfully amplified, whereas only a quarter of the newest specimens (1991–2008) were successful. The reasons for this unexpected result are unknown. One possibility for newer samples being less likely to successfully amplify AMF DNA is modern collection practices. We speculate that newer specimens may have been more likely to be dried using a heat source (e.g., drying ovens, cabinets, or space heaters) that inadvertently degraded AMF DNA, whereas older specimens might have been more likely to be air dried. Excessive heat has been found to lead to degraded DNA, with best preservation of DNA occurring under cool, dry conditions (Bollongino et al., 2008). Future protocol tests are needed to determine optimal collection practices for this use.

AMF community composition exhibited no effect of time period of collection. Replication within each species and 40‐year binned time periods inevitably limited our statistical power to detect a potential temporal signal. Given the sheer size and scope of herbaria worldwide (Thiers, 2018), specific global change hypotheses can be tested with careful specimen selection (e.g., effects of nitrogen deposition in northeastern U.S. hardwood forests), leveraging multiple herbaria (including duplicate specimens stored at multiple herbaria), and choosing species with adequate temporal and geographic representation. Furthermore, localities represented by historic specimens could be revisited to obtain contemporary specimens to compare to historic specimens.

### Can herbarium specimens be used to quantify AMF community differences between plant host species?

Although we did not detect significant AMF differences through time, our results show belowground communities were distinct between species. Based on prior studies in these plant species (e.g., Burke, 2008), we expected AMF communities to cluster by plant host species. Our results using herbarium samples collected from a decade to over a century ago were similar to a prior study on these species (Burke, 2008), with AMF communities signficantly different between plant host species. Nonetheless, visual examination of NMS ordination may suggest overlap in AMF communities between species, with *Arisaema* Mart. communities a possible subset of the community present in *Trillium* and *Maianthemum* F. H. Wigg. In many respects, this may not be surprising as both *Trillium* and *Maianthemum* have perennial root systems, with roots living for 5–7 years, whereas *Arisaema* has an annual root system with roots developing in spring and senescing in autumn (Brundrett and Kendrick, 1990a, 1990b). This pattern could explain the greater similarity in the root system of *Arisaema* samples within our study. In any case, our work shows that in addition to the ability for retrospective analyses, using herbarium specimens holds great promise as an efficient approach to compare AMF associations across many taxa worldwide. Additional methodological considerations may be needed in future studies of species with more complex root systems, such as standardizing specimen sampling through hierarchical root order–based and functional classification (McCormack et al., 2015).

### A novel, unanticipated role for herbaria in belowground ecology

Herbaria are becoming widely recognized as temporally and spatially extensive sources of genotypic, phenotypic, and biogeographic data (Heberling and Isaac, 2017), especially in the context of human‐induced environmental change (Lavoie, 2013). Enabled by relatively recent developments in molecular methods, herbarium specimens are being increasingly appreciated as a source for DNA for evolutionary analyses (Bieker and Martin, 2018). Unsurprisingly, genetic studies using plant specimens focus on plant DNA. Interestingly, however, one of the earliest genetic studies using herbarium material amplified DNA of the oomycete *Phytopthora infestans*, which was responsible for the Irish potato famine, from century‐old cultivated potato specimens (Ristaino et al., 2001). Natural history collections may unintentionally capture biological data outside of the target specimens. Using next‐generation DNA amplicon sequencing, Datlof et al. (2017) first reported the use of plant DNA banks to understand large‐scale patterns in fungal diversity in the phyllosphere that were unintentionally preserved in plant DNA samples. To our knowledge, the use of traditional herbarium specimen roots as a source of microbial DNA has not been tested before this study. Our approach introduces a new frontier for herbarium‐based genetic analyses.

Given that herbarium specimens were not collected and preserved for this specific use, a major concern in the present study was the potential confounding effect of specimen contamination. We controlled for fungal contamination from external sources through a root washing procedure and confirmed low contamination through comparing leaf (which should not contain AMF) and root tissue samples. This approach should be considered conservative, as root washing likely removed additional ecologically relevant AMF DNA in the rhizosphere. Future studies should test whether controlling for contamination is necessary and, if so, consider alternative approaches to address or prevent contamination.

Our use of herbarium specimen roots highlights new considerations in herbarium practices. It is standard protocol for plant collectors to include as much of the plant as possible, including belowground structures (Bridson and Forman, 1998). The primary reason for including belowground structures and roots with specimens of herbaceous species was for identification purposes, as these structures may help to determine the identity of some taxa (Fogg, 1940). However, most specimens do not include roots, especially more recent specimens. For the four focal species in the current study, we surveyed all specimens from Pennsylvania in the Carnegie Museum herbarium (CM). Only a minority of these specimens included roots: *A. triphyllum*, 26% (161/614); *M. racemosum*, 20% (94/467); *T. erectum*, 28% (131/460); and *T. grandiflorum*, 26% (82/319).

There are three possible reasons botanists may not frequently collect belowground tissue. First, it can be logistically difficult or time consuming to remove the belowground structures and clean them of excess soil, and this adds bulk that makes plant pressing, drying, and mounting to herbarium sheets more difficult. Second, collectors might intentionally collect only aboveground tissue for ethical reasons. There are serious concerns for the future of many populations of these understory species (Whigham, 2004), and by leaving their belowground storage organs and roots intact, these herbaceous perennials can return the following year. Last, root traits are used in the identification of only a minority of species, so collecting roots may simply not be perceived as useful for many taxa. Although both logistical and ethical considerations are necessary, our study highlights the importance of including belowground structures in newly collected specimens and establishing standard collection and preservation protocols. In addition to traditional vouchers, we recommend plant collectors consider also collecting roots in silica gel, similar to what is being done with leaf material for DNA analysis (e.g., Funk et al., 2017). We also suggest that collection databases include a new field to indicate whether a specimen contains roots or not. Such data will help researchers more easily identify which specimens are appropriate for root analyses. Similar protocols have recently been developed to score reproductive phenology of herbarium specimens (Yost et al., 2018).

Our results demonstrate an innovative, new use for herbaria in belowground biology, especially in the context of global change. This approach could be extended to other AMF‐dependent species, ecosystems (e.g., grasslands), and additional types of mycorrhizae (e.g., orchids). Microbial DNA in herbarium specimen roots have the potential to improve our understanding of basic belowground ecology, the implications of global change, and the conservation of threatened species. Although our study was limited in geographic and taxonomic scope, this approach has wider applications for microbial ecology, conservation biology, invasion biology, and evolutionary biology.

## Supporting information


**APPENDIX S1.** DNA quality for *Maianthemum racemosum* herbarium root samples. Samples are flanked by DNA markers that indicate size. Invitrogen Low DNA Mass Ladder is on the left and λ/*Eco*R1 + *Hin*dIII ladder is on the right (Thermo Fisher Scientific), with fragment sizes shown. ID numbers indicate the last three digits of the specimen ID number (see Appendix 1). Year refers to year of collection. Fifteen percent of the DNA extract was loaded into the 2% agarose gel using TAE buffer. Samples with degraded DNA are revealed by smearing in lane and did not amplify well with PCR. Labels underlined in blue indicate samples with successful arbuscular mycorrhizal fungi (AMF) DNA amplification in roots. Labels in red indicate samples that were not included in analyses either because roots failed to amplify or leaf TRFLP profiles suggested contamination.

**APPENDIX S2.** PCR results for *Arisaema triphyllum* samples as run into a 2% agarose gel in TAE buffer. Samples are flanked by DNA markers that indicate size. Invitrogen Low DNA Mass Ladder is on the left and λ/*Eco*R1 + *Hin*dIII ladder is on the right (Thermo Fisher Scientific), with fragment sizes shown. NC = negative control, +C = positive control arbuscular mycorrhizal fungi (AMF) plasmid. ID numbers indicate the last three digits of the specimen ID number (see Appendix 1). Year refers to year of collection. Labels underlined in blue indicate samples with successful AMF DNA amplification in roots. Labels in red indicate samples that were not included in analyses either because roots failed to amplify or leaf TRFLP profiles suggested contamination.

**APPENDIX S3.** PCR results for *Maianthemum racemosum* samples as run into a 2% agarose gel in TAE buffer. Samples are flanked by DNA markers that indicate size. Invitrogen Low DNA Mass Ladder is on the left and λ/*Eco*R1 + *Hin*dIII ladder is on the right (Thermo Fisher Scientific), with fragment sizes shown. NC = negative control, +C = positive control arbuscular mycorrhizal fungi (AMF) plasmid. ID numbers indicate the last three digits of the specimen ID number (see Appendix 1). Year refers to year of collection. Labels underlined in blue indicate samples with successful AMF DNA amplification in roots. Labels in red indicate samples that were not included in analyses either because roots failed to amplify or leaf TRFLP profiles suggested contamination.

**APPENDIX S4.** Results from alternative community analysis using a data set with single *Trillium grandiflorum* sample removed. Summary statistics for two‐way permutational multivariate ANOVA (PERMANOVA) of arbuscular mycorrhizal fungal communities among host plant species. The Bray–Curtis distance metric was used to calculate dissimilarity matrix.

## Data Availability

Recovered sequences from this study were deposited in GenBank (accession numbers MH800915–MH800957).

## APPENDIX 1 List of herbarium specimens sampled from the Carnegie Museum of Natural History Herbarium (CM), Pittsburgh, Pennsylvania, USA.

**Table:**

Species	Sheet no.	County^a^	Primary collector (no.)	Year	PCR result^b^
*Arisaema triphyllum* L.	CM024222	Westmoreland	Shafer, J. A. (s.n.)	1882	Positive
	CM024210	Allegheny	Shafer, J. A. (s.n.)	1900	Positive
	CM024423	Fayette	Jennings, O. E. (s.n.)	1905	Positive
	CM024168	Fayette	Jennings, O. E. (s.n.)	1917	—
	CM024186	Washington	Henry, L. K. (s.n.)	1940	Positive
	CM024438	Westmoreland	Henry, L. K. (s.n.)	1945	—
	CM024269	Washington	Buker, W. E. (s.n.)	1967	—
	CM024151	Allegheny	Buker, W. E. (s.n.)	1968	Positive
	CM301935	Westmoreland	Leberman, R. C. (s.n.)	1983	Positive
	CM389892	Westmoreland	Utech, F. H. (94‐246)	1994	—
	CM500379	Westmoreland	Isaac, B. L. (14427)	2002	—
	CM512500	Washington	Speedy, L. (LSB243)	2005	—
*Mainthemum racemosum* (L.) Link	CM029420	Beaver	Andriessen, H. (s.n.)	1888	Positive
	CM029400	Allegheny	Jennings, O. E. (s.n.)	1904	Positive
	CM029283	Fayette	Jennings, O. E. (s.n.)	1905	Positive
	CM029503	Allegheny	Slease, A. (s.n.)	1916	Positive
	CM029362	Westmoreland	Van Dersal, W. R. (s.n.)	1930	—
	CM029418	Beaver	Boardman, C. M. (s.n.)	1932	Positive
	CM029564	Clarion	Henry, L. K. (s.n.)	1960	—
	CM263733	McKean	Utech, F. H. (72‐008)	1972	Positive
	CM331052	Fayette	Utech, F. H. (87‐147)	1987	—
	CM397373	Fayette	Isaac, B. L. (7079)	1995	—
	CM478111	Westmoreland	Utech, F. H. (97‐282)	1997	—
	CM515067	Indiana	Speedy, L. (LS‐07‐354)	2007	—
*Trillium erectum* L.	CM110595	Westmoreland	Block, P. D. (s.n.)	1881	Positive
	CM190123	Allegheny	Shafer, J. A. (s.n.)	1883	—
	CM190128	Allegheny	Jennings, O. E. (s.n.)	1908	Positive
	CM023119	Allegheny	Jennings, O. E. (s.n.)	1912	—
	CM190119	Allegheny	Ingram (s.n.)	1919	Positive
	CM190185	Fayette	Graham, E. H. (s.n.)	1934	—
	CM041761	Westmoreland	Haywood, M. J. (s.n.)	1969	Positive
	CM306629	Allegheny	Behrer, B. (LSCW 29)	1984	Positive
	CM329455	Westmoreland	Utech, F. H. (87‐008)	1987	Positive
	CM361934	Allegheny	Zanol, W. A. (442)	1991	Positive
	CM468172	Westmoreland	Speedy, L. (08‐0190)	2008	Positive
	CM516701	Westmoreland	Speedy, M. L. (08‐0067)	2008	—
*Trillium grandiflorum* (Michx.) Salisb.	CM030163	Allegheny	Shafer, J. A. (s.n.)	1884	—
	CM030159	Allegheny	Shafer, J. A. (s.n.)	1899	—
	CM030156	Allegheny	Jennings, O. E. (s.n.)	1905	—
	CM030172	Allegheny	Jennings, O. E. (s.n.)	1915	—
	CM030178	Allegheny	Gibson, M. (s.n.)	1916	—
	CM030197	Westmoreland	Jennings, O. E. (s.n.)	1916	—
	CM263589	Westmoreland	Utech, F. H. (78‐089)	1979	—
	CM306393	Washington	Nishida, J. H. (619)	1984	—
	CM314708	Washington	Thompson, S. A. (2258)	1985	—
	CM512478	Washington	Speedy, L. (LSB270)	2005	—
	CM513677	Washington	Speedy, L. (LSB204)	2005	Positive
	CM468171	Westmoreland	Speedy, L. (08‐0191)	2008	—

a All specimens were collected in western Pennsylvania.

b ”Positive” refers to good amplification and included in analyses; “—” refers to poor amplification.
