# Supplementary material for: Utilizing herbarium specimens to quantify historical mycorrhizal communities
Source: Appl Plant Sci. 2019 Feb 28;7(4):e01223. doi: 10.1002/aps3.1223 (PMC6476165; doi:10.1002/aps3.1223)

Appendix S1. DNA quality for *Maianthemum racemosum* herbarium root samples. Samples are flanked by DNA markers that indicate size. Invitrogen Low DNA Mass Ladder is on the left and λ/*Eco*R1 + *Hin*dIII ladder is on the right (Thermo Fisher Scientific), with fragment sizes shown. ID numbers indicate the last three digits of the specimen ID number (see Appendix 1). Year refers to year of collection. Fifteen percent of the DNA extract was loaded into the 2% agarose gel using TAE buffer. Samples with degraded DNA are revealed by smearing in lane and did not amplify well with PCR. Labels underlined in blue indicate samples with successful arbuscular mycorrhizal fungi (AMF) DNA amplification in roots. Labels in red indicate samples that were not included in analyses either because roots failed to amplify or leaf TRFLP profiles suggested contamination.

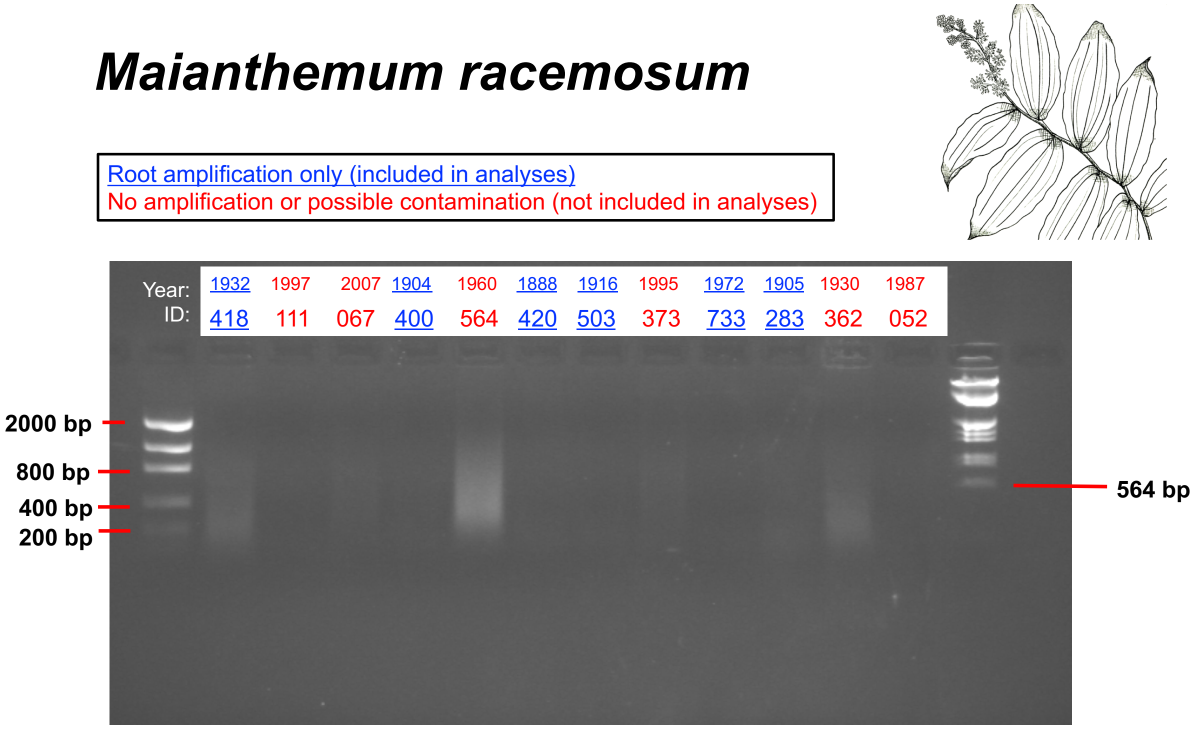

Supplement: Supplementary file 1 — APPENDIX S1. DNA quality for Maianthemum racemosum herbarium root samples. Samples are flanked by DNA markers that indicate size. Invitrogen Low DNA Mass Ladder is on the left and λ/EcoR1 + HindIII ladder is on the right (Thermo Fisher Scientific), with fragment sizes shown. ID numbers indicate the last three digits of the specimen ID number (see Appendix 1). Year refers to year of collection. Fifteen percent of the DNA extract was loaded into the 2% agarose gel using TAE buffer. Samples with degraded DNA are revealed by smearing in lane and did not amplify well with PCR. Labels underlined in blue indicate samples with successful arbuscular mycorrhizal fungi (AMF) DNA amplification in roots. Labels in red indicate samples that were not included in analyses either because roots failed to amplify or leaf TRFLP profiles suggested contamination. [file APS3-7-e01223-s001.pdf]
