# Supplementary material for: Utilizing herbarium specimens to quantify historical mycorrhizal communities
Source: Appl Plant Sci. 2019 Feb 28;7(4):e01223. doi: 10.1002/aps3.1223 (PMC6476165; doi:10.1002/aps3.1223)

Appendix S3. PCR results for *Maianthemum racemosum* samples as run into a 2% agarose gel in TAE buffer. Samples are flanked by DNA markers that indicate size. Invitrogen Low DNA Mass Ladder is on the left and λ/*Eco*R1 + *Hin*dIII ladder is on the right (Thermo Fisher Scientific), with fragment sizes shown. NC = negative control, +C = positive control arbuscular mycorrhizal fungi (AMF) plasmid. ID numbers indicate the last three digits of the specimen ID number (see Appendix 1). Year refers to year of collection. Labels underlined in blue indicate samples with successful AMF DNA amplification in roots. Labels in red indicate samples that were not included in analyses either because roots failed to amplify or leaf TRFLP profiles suggested contamination.

Root amplification only (included in analyses)
No amplification or possible contamination (not included in analyses)

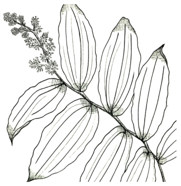

Maianthemum racemosum

A) Roots

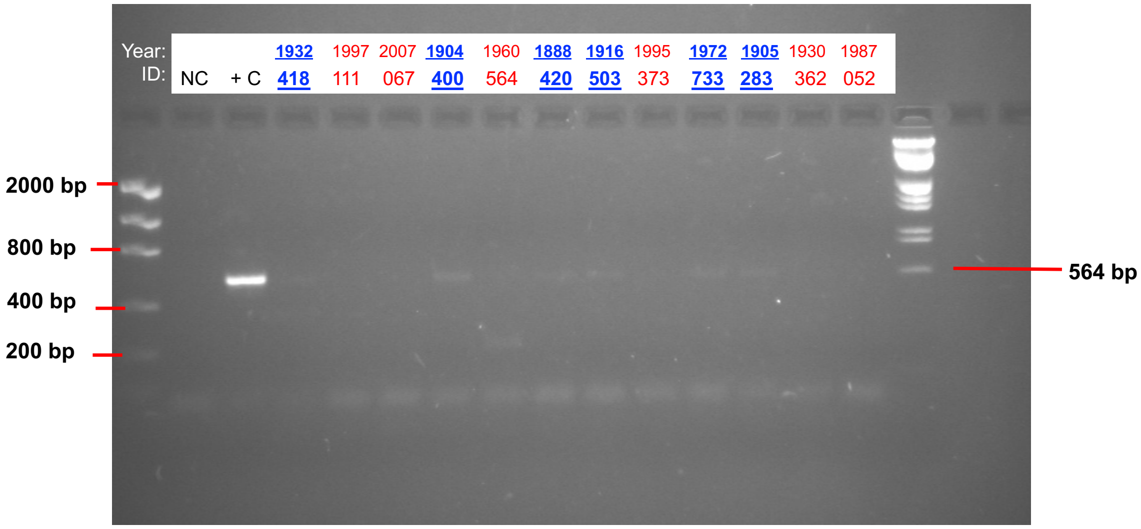

B) Leaves

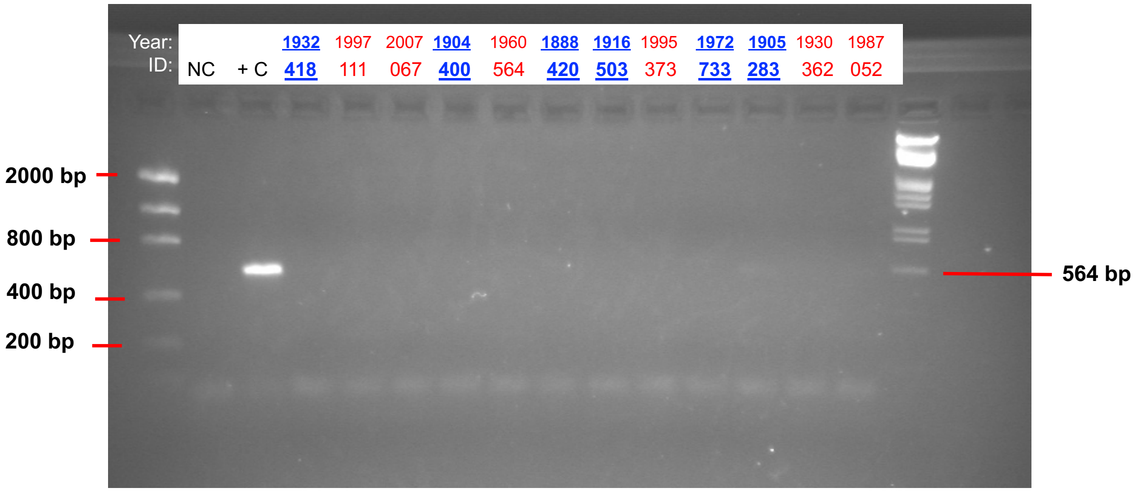

Supplement: Supplementary file 3 — APPENDIX S3. PCR results for Maianthemum racemosum samples as run into a 2% agarose gel in TAE buffer. Samples are flanked by DNA markers that indicate size. Invitrogen Low DNA Mass Ladder is on the left and λ/EcoR1 + HindIII ladder is on the right (Thermo Fisher Scientific), with fragment sizes shown. NC = negative control, +C = positive control arbuscular mycorrhizal fungi (AMF) plasmid. ID numbers indicate the last three digits of the specimen ID number (see Appendix 1). Year refers to year of collection. Labels underlined in blue indicate samples with successful AMF DNA amplification in roots. Labels in red indicate samples that were not included in analyses either because roots failed to amplify or leaf TRFLP profiles suggested contamination. [file APS3-7-e01223-s003.pdf]
