# Supplementary material for: Utilizing herbarium specimens to quantify historical mycorrhizal communities
Source: Appl Plant Sci. 2019 Feb 28;7(4):e01223. doi: 10.1002/aps3.1223 (PMC6476165; doi:10.1002/aps3.1223)
Supplement: Supplementary file 4 — APPENDIX S4. Results from alternative community analysis using a data set with single Trillium grandiflorum sample removed. Summary statistics for two‐way permutational multivariate ANOVA (PERMANOVA) of arbuscular mycorrhizal fungal communities among host plant species. The Bray–Curtis distance metric was used to calculate dissimilarity matrix. [file APS3-7-e01223-s004.docx]

**Appendix S4.** Results from alternative community analysis using a data set with single *Trillium grandiflorum* sample removed. Summary statistics for two-way permutational multivariate ANOVA (PERMANOVA) of arbuscular mycorrhizal fungal communities among host plant species. The Bray–Curtis distance metric was used to calculate dissimilarity matrix.

| **Source of variation** | ***df*** | **SS** | **MS** | ***F*** | ***P*** |
| --- | --- | --- | --- | --- | --- |
| Species | 1 | 0.3781 | 0.3781 | 2.462 | 0.0476* |
| Time | 1 | 0.1104 | 0.1104 | 0.719 | 0.6162 |
| Species × Time | 1 | 0.1713 | 0.1713 | 1.115 | 0.3656 |
| Residuals | 16 | 2.4578 | 0.1536 |  |  |
| Total | 19 | 3.1177 |  |  |  |

*Note: df* = degrees of freedom; *F* = test statistic; MS = mean squares; SS = sum of squares.

*Significant results (*P* < 0.05).
